# Assessment of Neuropsychological Function in Veterans With Blast-Related Mild Traumatic Brain Injury and Subconcussive Blast Exposure

**DOI:** 10.3389/fpsyg.2021.686330

**Published:** 2021-06-28

**Authors:** Ashley N. Clausen, Heather C. Bouchard, VA Mid-Atlantic MIRECC Workgroup, Kathleen A. Welsh-Bohmer, Rajendra A. Morey

**Affiliations:** ^1^Kansas City VA Medical Center, Kansas City, MO, United States; ^2^Duke-University of North Carolina at Chapel Hill Brain Imaging and Analysis Center, Duke University, Durham, NC, United States; ^3^VA Mid-Atlantic Mental Illness Research, Education and Clinical Center (MIRECC), Durham Veteran Affairs Healthcare System, Durham, NC, United States; ^4^Department of Psychiatry and Behavioral Sciences, Duke University, Durham, NC, United States; ^5^Center for Cognitive Neuroscience, Duke University, Durham, NC, United States

**Keywords:** subconcussive, traumatic brain injury, neuropsychological function, military, cognition, head injuries, blast concussion

## Abstract

**Objective:** The majority of combat-related head injuries are associated with blast exposure. While Veterans with mild traumatic brain injury (mTBI) report cognitive complaints and exhibit poorer neuropsychological performance, there is little evidence examining the effects of subconcussive blast exposure, which does not meet clinical symptom criteria for mTBI during the acute period following exposure. We compared chronic effects of combat-related blast mTBI and combat-related subconcussive blast exposure on neuropsychological performance in Veterans.

**Methods:** Post-9/11 Veterans with combat-related subconcussive blast exposure (*n* = 33), combat-related blast mTBI (*n* = 26), and controls (*n* = 33) without combat-related blast exposure, completed neuropsychological assessments of intellectual and executive functioning, processing speed, and working memory via NIH toolbox, assessment of clinical psychopathology, a retrospective account of blast exposures and non-blast-related head injuries, and self-reported current medication. Huber Robust Regressions were employed to compare neuropsychological performance across groups.

**Results:** Veterans with combat-related blast mTBI and subconcussive blast exposure displayed significantly slower processing speed compared with controls. After adjusting for post-traumatic stress disorder and depressive symptoms, those with combat-related mTBI exhibited slower processing speed than controls.

**Conclusion:** Veterans in the combat-related blast mTBI group exhibited slower processing speed relative to controls even when controlling for PTSD and depression. Cognition did not significantly differ between subconcussive and control groups or subconcussive and combat-related blast mTBI groups. Results suggest neurocognitive assessment may not be sensitive enough to detect long-term effects of subconcussive blast exposure, or that psychiatric symptoms may better account for cognitive sequelae following combat-related subconcussive blast exposure or combat-related blast mTBI.

## Introduction

For post-9/11 military service members, the majority of combat-related mild traumatic brain injuries (mTBI) are associated with blast exposure (Galarneau et al., 2008; Greer et al., 2016). Nearly 50% of service members report subconcussive blast exposure (Hoge et al., 2008), which is defined as exposure to a blast that does not elicit symptoms of mTBI (i.e., acute post-concussive symptoms). Blast-exposures can cause injury through a combination of mechanisms: over pressurization, impacts from projectiles or significant debris, forceful impacts against solid objects, or from subsequent related events (e.g., radiation burns, toxic chemicals; Taber et al., 2006; Burgess et al., 2010). Acute and chronic effects of blast-related injuries are difficult to assess given the heterogeneity in preinjury variables (e.g., genetic polymorphisms and/or premorbid psychiatric conditions), exposure during injury (e.g., distance from impact, repeated exposure, and when the injury is formally assessed and diagnosed), post-injury factors such as the military culture of underreporting mTBI symptoms and avoiding treatment, and the aging process, which may be influenced by blast-exposure (Bryden et al., 2019; Jorgensen-Wagers et al., 2021).

A unique constellation of sequelae has been associated with blast-exposure including alterations within the brain, somatic and cognitive complaints, and increased rates of comorbid psychiatric diagnoses (Clark et al., 2009; Belanger et al., 2011; Miller et al., 2016; Riedy et al., 2016). Studies have identified acute deficits from military-related mTBI (frequently resulting from blast exposure) associated with processing speed, verbal and visual memory, executive function, and reaction time (Luethcke et al., 2011; Kontos et al., 2013; Karr et al., 2014; Norris et al., 2014; Spira et al., 2014; Pagulayan et al., 2018). However, research on subconcussive blast exposure is mixed. There is some evidence of a relationship between blast exposure and measures of cognitive function including memory (Tate et al., 2013; Carr et al., 2016; Grande et al., 2018), and reaction time (Tate et al., 2013; Haran et al., 2019; LaValle et al., 2019). However, another study examining blast-exposure in police officers was unable to identify a significant relationship between blast-exposure and neuropsychological functioning (Baker et al., 2011). Neuroimaging research exploring primary blast exposure indicates evidence that both concussive (mTBI) and subconcussive blast exposure are associated with alterations in brain function within the default mode and dorsal attention networks (Robinson et al., 2015, 2017), functional connectivity within the default mode network (Robinson et al., 2015), and white matter damage associated with lower fractional anisotropy and higher radial diffusivity (Taber et al., 2015; Trotter et al., 2015), which may explain cognitive deficits associated with blast exposure.

Studies examining the role of psychiatric symptoms indicate neuropsychological sequelae observed in blast exposed Veterans may be better explained by psychiatric symptoms, specifically PTSD, as opposed to blast exposure (Mac Donald et al., 2014; Storzbach et al., 2015; Mattson et al., 2019; Nelson et al., 2020). More recently, an investigation of Veterans with blast exposure and PTSD indicated poor cognitive outcomes may result from psychiatric symptoms and/or aspects of the blast itself such as intensity as opposed to blast exposure (Martindale et al., 2020). Equivocal findings highlight the level of difficulty in examining the unique effects of blast-exposure in military and Veteran populations. Therefore, although blast exposure with and without mTBI may be linked with specific aspects of cognition including measures of executive functioning, processing speed, and reaction time, it remains difficult to discern the impact of psychiatric symptoms vis-à-vis TBI on neuropsychological functioning.

The present study aimed to explore possible differences in neuropsychological functioning in Veterans with combat-related blast mTBI, combat-related subconcussive blast exposure, and controls who did not experience a combat-related blast exposure. Based on previous findings (Robinson et al., 2015; Taber et al., 2015; Trotter et al., 2015; Carr et al., 2016), we hypothesized Veterans with combat blast-related mTBI and Veterans with combat subconcussive blast exposure would demonstrate significantly slower processing speed compared to Veterans without combat blast exposure. We controlled for the effects of PTSD on the relationships between type of head injury (combat-related blast mTBI, subconcussive, and controls) and neuropsychological performance. We hypothesized Veterans in the mTBI and subconcussive groups would continue to exhibit slower processing speed compared with the control group when controlling for PTSD. We also explored neuropsychological domains of executive functioning, inhibition, working memory. Based on previous findings, we hypothesized that the combat-related blast mTBI group would exhibit poorer executive function and working memory relative to the control group.

## Methods

### Participants

The present study included 96 Veterans. Participants were recruited between 2015 and 2019 from the VA Mid-Atlantic MIRECC post-deployment mental health repository (Brancu et al., 2017). Participants underwent screening for inclusion and exclusion criteria based on information available in the repository and subsequent telephone screenings. Male and female Veterans were included in the present study if they were between the ages of 18–65, served during or after 9/11/2001, fluent in English, capable of providing informed consent, and not constrained from participating over the duration of the study in all study activities. Participants were excluded from the current study if they met criteria for current or lifetime substance dependence (except nicotine), Axis I psychiatric disorders (except PTSD, Generalized Anxiety Disorder, Panic Disorder, Agoraphobia, Other specific phobias, Anxiety not otherwise specified, and Depression) assessed using the Structured Clinical Interview for DSM-IV Axis I Disorders (SCID; First et al., 1996). The parent study aimed to explore the relationship between blast exposure and cognitive functioning with brain structure using diffusion imaging to assess white matter health and resting state functional MRI to examine functional connectivity (P.I. Morey, R.A.). As such, participants were also excluded from the present analysis if they had been clinically diagnosed neurological conditions such as multiple sclerosis, stroke, seizure disorder, etc., or if they had prior neurosurgery, lesions on conventional magnetic resonance imaging (MRI), and/or pregnant. Notably, given the nature of the current research, Veterans with a history of mTBI were included in the present study. Research was completed in accordance with Helsinki Declaration, and all participants provided written informed consent. Study procedures were approved by the Institutional Review Boards at Duke University Medical Center and Durham VA Medical Center. Neuroimaging data from participants in the present study are included in our published manuscript (Clausen et al., 2020) but the corresponding neurocognitive data has not been published previously.

### Neuropsychological Assessment

Veterans completed a brief assessment of neuropsychological functioning using the National Institutes of Health (NIH) Toolbox Cognition Battery (see nihtoolbox.org) (Hodes et al., 2013), which compares favorably to traditional gold standard measures on convergent and discriminant validity (Weintraub et al., 2014; Zelazo et al., 2014), and been used to assess neuropsychological functioning in military populations (Walker et al., 2016; Dunbar et al., 2019). Based on findings from previous research, we focused our assessment of neuropsychological functioning on domains previously identified in relation to mTBI and PTSD including assessment of intelligence (IQ), processing speed (Pattern Comparison), executive function (Dimensional Change Card Sort), attention and response inhibition (Flanker Inhibitory Control and Attention), and working memory (List Sorting Working Memory). All tasks were completed on an iPad with iOS running the NIH Toolbox. T-scores were calculated from raw scores on each subtest and were adjusted for age and education. Veterans completed the Wechsler Test of Adult Reading to estimate premorbid intellectual abilities (IQ; Venegas and Clark, 2011), and the Test of Memory Malingering (TOMM), a visual recognition task, to gauge effort during testing and to ensure validity of neuropsychological assessment (Tombaugh, 1997). Scores ≤ 44 on trial 2 of the TOMM are suggestive of poor effort (Tombaugh, 1997).

### TBI Assessment

The Quantification of Cumulative Blast Exposure (QCuBE) was used to assess the presence and severity of blast-related TBI (Petrie et al., 2014). The QCuBE is a semi-structured interview, administered by trained research personnel, assessing the number and severity of blast-exposures. The QCuBE provides a detailed assessment in order of severity of up to five individual blast exposures including device type, tamping forces, distance from the blast, context of the explosion, and immediate neurobehavioral symptoms following blast exposure. In addition, lifetime exposure of non-blast-related head trauma is recorded from incidents such as assaults, sports injuries, falls, and motor vehicle accidents. Four Veterans did not complete the QCuBE. Thus, a total of 92 Veterans were categorized into three groups based only on combat-related blast exposure including (1) blast-unexposed (*n* = 33), who report no exposure to combat-related blasts (control group), (2) blast-exposed (*n* = 33) with self-reported exposure only to blast forces occurring during combat, and either no acute symptoms or insufficient symptoms to meet criteria for mTBI (subconcussive group), and (3) blast mTBI (*n* = 26) who reported exposure to combat blast forces accompanied by symptoms consistent with mTBI (blast mTBI group) based on criteria established by the American College of Rehabilitation Medicine (ACRM; Head, 1993) and the Departments of Veteran Affairs and Defense (Management of Concussion/mTBI Working Group, 2009). Groups exposed to combat blasts (subconcussive vs. blast mTBI) were differentiated based on previously published diagnostic categories (Walker et al., 2015) including the presence or absence of loss of consciousness, amnesia/memory loss, feeling dazed or confused, and whether the participant reported their head hit significant objects/debris. Notably, individuals in all groups, including the control group, may have been exposed to non-combat blast exposure during training exercises, or prior mTBI. Individuals in the control group included those with and without combat deployments. Only three participants in the control group denied combat and non-combat related head injuries and/or blast-exposure. Blast exposure was inconsistently reported across participants, with some participants providing a “greater than” number (e.g., “>6”), some provided estimates (e.g., “~50”), while others provided a numeric value (e.g., “6”). To quantify number of exposures, the response was converted to the root number provided (i.e., “>6” was converted to the numerical value “6”). Thus, our analyses focused on the presence or absence of blast exposure, and presence or absence of post-concussive symptoms. To better understand the unique impact of blast-exposure, the number of lifetime non-blast head injuries was included as a covariate in the main analyses.

### Clinical Assessment

Current PTSD symptom severity was assessed using the Clinician Administered PTSD Scale (CAPS)-−5 (Weathers et al., 2013, 2018), which is a clinician-administered interview, administered by trained research personnel. Veterans completed self-report questionnaires to assess depression, alcohol use, and combat exposure including the Beck Depression Inventory-II (BDI–II; Beck et al., 1996), the Alcohol Use Disorders Identification Test (AUDIT; Saunders et al., 1993), and the Combat Exposure Scale (CES; Keane et al., 1989).

Demographic information, current psychotropic medication, and history of neurodevelopmental disorders including learning disabilities and attention deficit and hyperactivity disorder (ADHD), was self-reported. Highest level of education was assessed categorically. A score of 0 indicates elementary education, 1 indicates a General Education Diploma (GED), 2 indicates high school diploma, 3 indicates technical or trade school, 4 indicates associates degree, 5 represents a bachelor's degree, 6 represents a master's or doctoral degree.

### Statistical Analysis

All statistical analyses were conducted in R Statistical Software. Sex, premorbid IQ, psychotropic medication use, PTSD, depressive, and alcohol use symptoms were explored as covariates using robust (Huber Robust Regression; MASS package; rlm command) analysis of variance (ANOVA) and chi-squared test. Next, we examined relationships between mTBI, subconcussive, and control groups on measures of neuropsychological functioning with robust analysis of covariance (ANCOVA), controlling for relevant demographic and clinical variables (rlm and ANOVA commands). Prior research investigating the effects of TBI has inconsistently included clinical covariates. Therefore, to make parallel comparisons, we examined relationships between blast exposure and neuropsychological function with and without clinical variables (i.e., PTSD symptoms). *Post-hoc* analyses to compare group means of ANCOVA analyses were conducted using Tukey HSD *post-hoc* analyses (glht command). To limit the inflation of Type I error due to multiple comparisons, FDR correction of Benjamini and Hochberg (Yoav and Hochberg, 1995) was applied to ANCOVAs for each neuropsychological domain, resulting in three separate comparisons. *Post-hoc* analyses were also FDR corrected for the number of group comparisons (*n* = 3 for each *post-hoc* analysis). Results were considered statistically significant at FDR-corrected *p* < 0.05.

## Results

Veterans ranged in age from 25 to 62 years (mean age = 44.8, *SD* = 9.4). The majority of Veterans were male (76%). Twenty-five Veterans were currently taking psychotropic medication. Average number of years since the worst blast exposure was 13.2 years (*SD* = 9.2). One Veteran reported exposure to 1,000 blasts. Given that this report was >4 standard deviations from the mean, this outlier was removed from the analysis related to number of blast exposures. There was a significant difference in number of reported exposures groups [*F*_(2, 88)_ = 8.62, *p* < 0.001]. Veterans in the mTBI ($\hat{\psi }$ = 3.6, *p* < 0.001) and subconcussive ($\hat{\psi }$ = 2.3, *p* = 0.044) groups exhibited significantly more blast exposure compared to Veterans in the control group. No differences were observed between Veterans in the mTBI and subconcussive groups ($\hat{\psi }$ = 1.3 *p* = 0.293). Performance on trial 2 of the TOMM indicated all Veterans put forth good effort (TOMM Trial 2 range 48-50). Demographic and clinical information is presented in Table 1.

**Table 1:** Sample demographics.

	**Full sample (*N* = 92)**	**Blast mTBI (*n* = 26)**	**Subconcussive (*n* = 33)**	**Controls (*n* = 33)**
	**Mean (*****SD*****)**	**Mean (*****SD*****)**	**Mean (*****SD*****)**	**Mean (*****SD*****)**
Age	44.8 (9.4)	43.3 (7.8)	43.8 (9.8)	47.0 (9.9)
**Education (%)**				
Technical or Trade School	17.4% (*n* = 16)	15.4% (*n* = 4)	15.2% (*n* = 5)	21.2% (*n* = 7)
Associates degree	26.1 (*n* = 24)	38.5% (*n* = 10)	18.2% (*n* = 6)	24.2% (*n* = 8)
Bachelor's degree	32.6% (*n* = 30)	26.9% (*n* = 7)	45.5% (*n* = 15)	24.2% (*n* = 8)
Master's or Doctoral degree	23.9% (*n* = 22)	19.2% (*n* = 5)	21.2% (*n* = 7)	30.3% (*n* = 10)
Gender (% male)	76.1% (*n* = 70)	84.6% (*n* = 22)	69.7% (*n* = 23)	75.8% (*n* = 25)
Psychotropic Medication	27.2% (*n* = 25)	42.3% (*n* = 11)	30.0% (*n* = 10)	12.1% (*n* = 4)
Years Since Worst Blast Exposure	13.2 (9.2)	12.2 (4.6)	12.6 (7.12)	13.8 (13.1)
Number of Blast Exposures	14.7 (41.7)	22 (58.7)	14.1 (33.0)	9.5 (32.5)
Number of Non-blast Head Injuries	2.0 (2.71)	3.08 (3.92)	1.70 (1.81)	1.45 (2.05)
Alcohol Use	3.0 (3.4)	4.6 (4.7)	3.0 (3.1)	2.7 (2.1)
Combat exposure severity	7.4 (6.7)	14.0 (5.8)	8.0 (4.8)	1.6 (2.7)
PTSD symptoms	12.4 (15.2)	15.7 (16.2)	9.1 (14.0)	2.8 (3.7)
Depressive symptoms	9.5 (9.9)	11.4 (10.1)	8.3 (10.3)	3.8 (5.7)
Premorbid IQ	109.6 (10.4)	106.1 (11.1)	109.4 (11.0)	112.8 (8.4)
Test of Effort (Trial 2)	49.9 (0.4)	49.9 (0.3)	49.8 (0.5)	50.0 (0.2)
Pattern Comparison (processing speed)	51.7 (12.4)	49.4 (12.5)	52.4 (10.4)	61.8 (11.0)
Dimensional Change Card Sort (executive functioning)	54.5 (12.7)	52.8 (11.7)	54.3 (15.4)	61.9 (10.6)
Flanker Inhibitory Control (Inhibition)	47.3 (9.3)	47.2 (9.6)	46.7 (8.9)	48.9 (8.6)
List Sorting Working Memory (working memory)	54.7 (10.1)	56.5 (9.7)	54.7 (13.3)	61.8 (7.3)

*mTBI, mild traumatic brain injury; SD, Standard Deviation; PTSD, post-traumatic stress disorder; IQ, intelligence quotient; chronicity of blast-exposure, duration in years since worst blast-exposure at time of neuropsychological assessment; PTSD symptoms were assessed using the Clinician Administered PTSD Scale 5; Depressive symptoms were assessed using the Beck Depression Inventory II; Premorbid IQ was assessed using the Wechsler Test of Adult Reading; Test of Effort was assessed using the Test of Memory Malingering; Processing speed, executive function, inhibitory function, and working memory were assessed using the NIH Toolbox Cognitive Battery*.

There were no differences between control, subconcussive and blast mTBI groups in age [*F*_(2, 89)_ = 1.3, *p* = 0.271], education [*F*_(2, 89)_ = 0.41, *p* = 0.666], neurodevelopmental disorders (χ^2^ = 0.89, *df* = 2, *p* = 0.639), premorbid IQ [*F*_(2, 88)_ = 3.11, *p* = 0.050], or alcohol use [*F*_(2, 89)_ = 2.97, *p* = 0.057]. While there was a larger proportion of males compared with females in the blast mTBI group (see Table 1), there were no significant differences in sex distribution between control, subconcussive and blast mTBI groups (χ^2^ = 3.95, *df* = 2, *p* = 0.138). Age, education, premorbid IQ, alcohol use, neurodevelopmental disorders, and sex were *not* included as covariates in the main analysis. Notably, T-scores derived from NIH toolbox were adjusted for age and education. Veterans in the blast mTBI group reported significantly more non-blast-related head injuries compared with controls ($\hat{\psi }$ = 1.3, *p* = 0.006). Significantly more Veterans in the blast mTBI group were prescribed psychotropic medication relative to the control group ($\hat{\psi }$ = 0.03, *p* = 0.03). Clinical covariates included the number of non-blast-related head injuries and psychotropic medication.

Severity of PTSD symptoms significantly differed between groups [*F*_(2, 28.16)_ = 7.02, *p* = 0.003]. Veterans in the blast mTBI group exhibited significantly higher PTSD symptom severity compared with the control group ($\hat{\psi }$ = −10.422, *p* = 0.0005), and the subconcussive group ($\hat{\psi }$ = −8.28, *p*=0.013). No differences were observed between the subconcussive and control groups on PTSD symptom severity ($\hat{\psi }$ = −2.14, *p* = 0.315). Differences in depressive symptoms were also observed between groups [*F*_(2, 23.06)_ = 6.05, *p* = 0.008], with Veterans in the blast mTBI group exhibiting more severe depressive symptoms compared with the control group ($\hat{\psi }\;=$ −7.19, *p* = 0.002). No differences were identified in depressive symptoms between control and subconcussive groups ($\hat{\psi }$ = −3.36, *p* = 0.216) nor subconcussive and blast mTBI groups ($\hat{\psi }$ = −3.84, *p* = 0.143). While depressive symptoms and PTSD symptoms were highly correlated in the present sample (*r* = 0.77, *p* < 0.001), the variance inflation factor (VIF) was <10 (VIF = 2.46) (O'Brien, 2007). Thus, both depressive and PTSD symptoms were retained in the analyses. Level of combat exposure significantly differed between groups [*F*_(2, 89)_ = 66.2, *p* < 0.001]. Veterans in the mTBI group endorsed significantly higher levels of combat exposure relative to the subconcussive ($\hat{\psi }$ = 6.3, *p* < 0.001) and control ($\hat{\psi }$ = 12.5, *p* < 0.001) groups. Veterans in the subconcussive group exhibited significantly higher combat compared to those in the control group ($\hat{\psi }$ = 6.3, *p* < 0.001). Therefore, supplemental analyses were then conducted to adjust for combat exposure. Akaike's Information Criteria (AIC) and Bayesian Information Criteria (BIC) indices were used to compare model fit. The model with the lowest AIC and BIC was identified as the model of best fit.

Omnibus ANCOVA results are displayed in Table 2. Differences between groups were identified on a measure of processing speed (*p* = 0.004, FDR corrected *p* = 0.016, Cohen's d = 0.43) and working memory (*p* = 0.023, FDR corrected *p* = 0.046, Cohen's d = 0.42). *Post-hoc* analyses revealed both mTBI ($\hat{\psi }$ = −12.43, *p* < 0.001, FDR corrected *p* = 0.003) and subconcussive ($\hat{\psi }$ = −10.02, *p* = 0.006, FDR corrected *p* = 0.009) groups exhibited slower processing speed compared with the control group. Notably, there were no differences between mTBI and subconcussive groups on measures of processing speed ($\hat{\psi }$ = −2.42, *p* = 0.728, FDR corrected *p* = 0.728). Related to working memory, *post-hoc* analyses revealed those in the blast mTBI group demonstrated lower scores on a working memory task compared with the control group. However, this difference did not reach the threshold for significance after FDR correction ($\hat{\psi }$ = −8.89, *p* = 0.044, FDR corrected *p* = 0.093). No differences were observed between mTBI and subconcussive groups $\hat{\psi }$ = −8.0, *p* = 0.062, FDR corrected *p* = 0.093) nor subconcussive and control groups $\hat{\psi }$ = −0.89, *p* = 0.964, FDR corrected *p* = 0.964). There were no differences between mTBI, subconcussive, and control groups on measures of executive functioning, or inhibition (all FDR corrected *p*'s > 0.070).

**Table 2:** Differences in neuropsychological functioning by group.

	***F***	***df***	***p***	**FDR corrected *p***	**Cohen's D**	**AIC/BIC**
**Blast mTBI, subconcussive blast, and control groups**						
Pattern Comparison (processing speed)	4.17	2, 85	0.004	0.016*	0.43	704.7/719.7
Dimensional Change Card Sort (executive functioning)	2.44	2, 85	0.053	0.071	0.33	714.9/723.9
Flanker Inhibitory Control (Inhibition)	1.14	2, 85	0.342	0.342	0.23	663.8/678.8
List Sorting Working Memory (working memory)	3.04	2, 66	0.023	0.046*	0.42	538.8/552.4
**Blast mTBI, subconcussive blast, and control groups adjusting for PTSD and depression**						
Pattern Comparison (processing speed)	3.16	2, 81	0.008	0.032*	0.38	688.3/705.7
Dimensional Change Card Sort (executive functioning)	2.08	2, 81	0.064	0.101	0.31	700.0/719.9
Flanker Inhibitory Control (Inhibition)	0.85	2, 81	0.538	0.538	0.20	651.5/671.3
List Sorting Working Memory (working memory)	2.02	2, 63	0.076	0.101	0.35	533.5/551.5

*mTBI, mild traumatic brain injury; blast-exposed, mild traumatic brain injury and subconcussive blast-exposed Veterans; PTSD symptoms were assessed using the Clinician Administered PTSD Scale 5; Depressive symptoms were assessed using the Beck Depression Inventory II; Premorbid IQ was assessed using the Wechsler Test of Adult Reading; Processing speed, executive function, inhibitory function, and working memory were assessed using the NIH Toolbox Cognitive Battery*.

When controlling for symptoms of PTSD and depression, in addition to prior non-blast-related head injuries and psychotropic medication use, we continued to observe a difference between mTBI, subconcussive, and control groups on a measure of processing speed (*p* = 0.008, FDR corrected *p* = 0.032, Cohen's d = 0.38). The blast mTBI group exhibited significantly slower processing speed compared with the control group ($\hat{\psi }$ = −10.40, *p* = 0.016, FDR corrected *p* = 0.048). No differences were identified between subconcussive and control groups ($\hat{\psi }$ = −2.27, *p* = 0.761, FDR corrected *p* = 0.076) or between mTBI and subconcussive groups ($\hat{\psi }$ = −8.12, *p* = 0.056, FDR corrected *p* = 0.076). When controlling for PTSD and depression, there were no differences between mTBI, subconcussive, and control groups on measures of executive functioning, inhibition, or working memory (all FDR corrected *p*'s > 0.101).

To further explore the relationship between processing speed and blast exposure, combat exposure was added as a covariate. The overall model predicting processing speed remained significant (*p* = 0.015); however, no significant differences were identified between mTBI, subconcussive and control groups (all *p*'s <0.072; all FDR corrected *p*'s <0.200). However, this model exhibited higher AIC and BIC indices (AIC = 690.3, BIC = 710.1) compared to the model controlling for PTSD and depressive symptoms (AIC = 688.3, BIC = 705.6). Therefore, the model controlling for psychiatric symptoms, but not combat exposure, was identified as the model of best fit.

## Discussion

While previous research has examined the acute effect of mTBI and subconcussive injuries on neuropsychological functioning, our study sought to explore long-term differences between Veterans with combat blast-related mTBI, combat subconcussive blast exposure, and controls without combat-related blast exposure. Further, we explored the potential effect of PTSD and depression on these relationships. We found the Veteran group with combat blast-related mTBI and the Veteran group with subconcussive blast exposure exhibited slower processing speed when compared with blast-unexposed Veteran controls before controlling for psychiatric symptoms. When accounting for PTSD and depressive symptoms, Veterans with mTBI continued to demonstrate slower processing speed compared with controls and a trend for slower processing speed in Veterans with subconcussive blast exposure compared with the blast mTBI group. However, this latter result did not reach the threshold for corrected statistical significance.

Previous research examining the effects of blast exposure on neuropsychological functioning has primarily focused on Veterans who met criteria for mTBI, and highlights a relationship between mTBI and deficits in processing speed, executive function, and verbal memory (Karr et al., 2014). These functions may improve over time, which suggests that distinguishing between acute vs. chronic effects of mTBI may be particularly important when examining neuropsychological functioning (Brenner et al., 2010; Dikmen et al., 2017). Consistent with previous findings, the present results support a relationship between blast-related mTBI and slower processing speed compared with controls. Notably, the mean time since most recent blast-exposure and neuropsychological assessment for the blast mTBI group was 10.2 years (*SD* = 4.8 years), allowing us to examine chronic effects of combat-related blast mTBI. Our findings highlight the relationship between mTBI and neuropsychological functioning, specifically processing speed, may be more chronic in duration than other cognitive constructs. Importantly, psychiatric conditions that are highly comorbid with mTBI including PTSD (Hoge et al., 2008; Morissette et al., 2011; Mac Donald et al., 2014), have also been associated with cognitive deficits (Aupperle et al., 2012; Trivedi and Greer, 2014) and are thought to mediate the relationship between head injury and cognitive sequelae (Nelson et al., 2020). However, similar to our findings with processing speed, a previous study reported a negative correlation between compromised white matter structure and processing speed in Veterans with TBI, but did not find a parallel association with PTSD (Sorg et al., 2016).

Research focusing on blast mTBI has provided considerable insights related to the acute and chronic effects of mTBI on neuropsychological functioning. However, nearly half of all service members report blast-exposure without post-concussive symptoms (Hoge et al., 2008), underscoring a critical need to evaluate the effects of subconcussive blast-exposure on neuropsychological functioning. While we found differences in processing speed between Veterans with subconcussive blast-exposure relative to controls, these findings were accounted for after controlling for PTSD, suggesting poorer performance was attributed to psychiatric symptoms rather than the presence of subconcussive exposure. Alternatively, while replication with a longitudinal dataset is needed, our results may indicate an interaction effect between subconcussive blast exposure and PTSD. The trend level finding may simply be due a lack of statistical power, which could be addressed with a larger sample. A small body of research has begun to examine these relationships and provides initial evidence that acute subconcussive injuries are correlated with intensity of blast exposure and associated with significant reductions in reaction time when compared with controls (Haran et al., 2019; LaValle et al., 2019). Thus, it is plausible our null results may be due to variations in distance to or intensity of blast exposure. Details of the blast exposure, including distance from blast, were inconsistently assessed in the present study, limiting our ability to examine whether distance impacted our results. However, current findings build on previous research and suggest that cognitive symptoms observed in chronic subconcussive blast exposure may be due to psychiatric symptoms.

### Limitations and Strengths

The present study provides a framework to examine differences between combat blast-related mTBI and subconcussive exposures, but it is not without limitations. The sample size of the blast-unexposed control Veterans is small despite concerted efforts to recruit Veterans who met the requisite inclusion and exclusion criteria, and thus may impact interpretation of current findings. This may be due to the increasing prevalence of explosions in the Afghanistan and Iraq conflicts. Currently, more than 60% of army infantry soldiers report more than one exposure to improvised explosive devices (Hoge et al., 2008), leading to a smaller fraction of Veterans without any self-reported blast exposure. Despite the small sample size of the unexposed control group, this limitation is partially addressed by the cognitive performance of the unexposed control group, which was consistent with the age adjusted population norms for the relevant neuropsychological tests. The number of blast exposures is difficult to accurately assess as repetitive exposures can occur in rapid succession during chaotic combat and training situations (Hoge et al., 2008; Carr et al., 2016), as well as occupational exposures that may occur outside of military service (e.g., police officers exposed to low-level blasts; Baker et al., 2011). This leads to difficultly in reliably parsing individual exposures. Even so, some published studies have found that the number of reported blast-related mTBIs or the proximity to blast did not affect symptom reporting (Lippa et al., 2010) or cognitive functioning (Ivanov et al., 2017). In addition, assessment of blast exposures relied on self-reported retrospective recall, which is prone subjective assessment of blast exposure, as well as false-positive and false-negative ascertainment, especially as elapsed time since the event increases (Van Dyke et al., 2010; Polusny et al., 2011), which limits the certainty of our group assignments. Effort during testing by Veterans seeking compensation for mTBI-related deficits can also vary (Denning and Shura, 2019), and while this context was not directly assessed, participants were evaluated using the TOMM and assured that our assessment was for research purposes only. Similarly, baseline neuropsychological measurements could not be assessed, nor could we completely parse deployment-specific effects such as PTSD. A number of other psychological, medical and environmental factors impact processing speed such as chronic pain, insomnia, and non-psychotropic medications but these were not assessed in the present sample. Lastly, neuropsychological assessment was completed using an iPad, which has been cited as a limitation by some experts but growing evidence suggests the iPad platform provides robust results (Rao et al., 2017; Bilder and Reise, 2019). iPad administration represents several strengths as it is more consistent from one rater to the next and from one site to the next, eliminates errors with data entry and scoring, allows for more accurate measurement of reaction timing, and allows for more robust inferences due to standardized administration and scoring procedures. Future longitudinal research with larger samples sizes in Veterans with and without psychiatric comorbidities and, chronic pain and more detailed information on blast history as well as medical history is warranted to characterize the causal relationships between blast-exposure and neuropsychological functioning.

## Conclusion

Prior research examining the effects of blast-exposure on neuropsychological function have primarily focused on those who sustained a mTBI, indicating the presence of persistent neurobehavioral sequelae. Our study examined both combat blast-related mTBI and subconcussive combat blast exposure in a single study and provides additional evidence that Veterans who sustain combat-related blast mTBI, as well as those who report subconcussive blast exposure, exhibit slower processing speed compared with blast-unexposed controls. Poorer performance on measures of processing speed remained when controlling for highly comorbid psychiatric symptoms of PTSD for those in the blast mTBI group, but not for those in the subconcussive group, suggesting a complex relationship between blast exposure and mental health symptoms on neuropsychological functioning, specifically processing speed.

## Data Availability Statement

The raw data supporting the conclusions of this article will be made available by the authors, without undue reservation.

## Ethics Statement

The studies involving human participants were reviewed and approved by Institutional Review Boards at Duke University Medical Center and Durham VA Medical Center. The patients/participants provided their written informed consent to participate in this study.

## Author Contributions

RM conceived and designed the research study, obtained study funding and study data, and revised and approved the final manuscript. AC developed the analytic plan, analyzed study data, drafted, and revised and approved the final manuscript. HB assisted with data collection, preliminary data analysis, and revised and approved the final manuscript. KW-B consulted on the study design and interpretation of the data analysis, and revised and approved the final manuscript. VA Mid-Atlantic MIRECC Workgroup contributors provided access to study data through the MIRECC repository, and revised and approved the final manuscript. All authors contributed to the article and approved the submitted version.

## Conflict of Interest

The authors declare that the research was conducted in the absence of any commercial or financial relationships that could be construed as a potential conflict of interest.
